# Proceedings of the Ninth Annual Meeting of the Tennessee Dental Association

**Published:** 1872-12

**Authors:** L. G. Noel


### PROCEEDINGS OF SOCIETIES.

### Proceedings of the Ninth Annual Meeting of the Tennessee Dental Association.

The Association met at the office of Dr. J. C. Ross, on Union street, Nashville, Oct 23, 1872, with the President, Dr. J. C. Ross, in the chair.

The meeting was called to oder by the President, and opened with prayer, by Dr. L. C. Chisholm.

The attendance was slim, only the following officers and members responding to roll call: President—J. C. Ross, Nashville; First Vice President, R. R. Freeman, Nashville; Second Vice President—A. F. Claywell, Lebanon; Recording Secretary—L. G. Noel, Nashville; W. H. Morgan, Nashville; E. A. Herman, Nashville; G. D. Buckner, Flat Creek; L. C. Chisholm, Nashville.

After going through with the usual preliminary bnsiness, such as report of Executive Committee, Committee of Arrangements, Committee on Transportation, etc., the various standing committees were called upon to report. Most of them were absent, and those present requested further time.

On motion of Dr. Morgan, the hours of meeting and adjournment were fixed as follows: Morning session from half past 9 to half past 12 o'clock; afternoon session from half past 2 to 4 o'clock; evening session from half past 7 to 10 o'clock.

Miscellaneous business being now in order, Dr. Chisholm opened the debate by the proposition that enamel is not designed by nature to protect the teeth from decay; but to resist the friction of mastication. He said teeth decay as readily where there is no defect in the enamel, when the circumstances are favorable to the retention of foreign substances, as in fissures and defective places. Decay almost always occurs where clasps are applied, because of the difficulty of securing cleanliness in these places. Enamel was intended to resist friction. If absolute cleanliness could be secured and always maintained, we would have little to fear from decay.

Dr. Morgan, asked Dr. Chisholm, to define the word cleanliness.

Dr. Chisholm—I mean to keep the mouth free at all times from decomposing foreign matter.

Dr. Morgan—I'll take a mouth in which there is no decomposing foreign matter at any time, and show you decay progressing rapidly from the action of vitiated saliva and mucus.

In measles and typhoid fever the fluids are always vitiated. In pregnacy decay always advances more rapidly than at any other time, particularly in defective places in the enamel. Its greater density and solidity enables it to resist the action of chemical agents physically better than the dentine with its greater vitality. The dentine is less dense, and affords more surface for the action of corrosive agents when exposed, and hence must be more liable to decay. I have been surprised by the position taken by Prof. Arthur, in regard to this question.

Dr. Chisholm—Dr. Morgan has not stated a single point that I cannot admit; but he cannot prove that the decay which takes place in fevers is not due to the presence of foreign matter, held in contact with the teeth by the viscidity of the fluids. I have never read Dr. Arthur's book, but I am glad to know there is some one to side with me in this matter, which I have made the study of years. I have never seen saliva so vitiated as to corrode the teeth, and must have a demonstration before I can accept it as true.

Dr. Morgan—I know by actual test that the saliva contains both sulphuric and lactic acid. Have seen in typhoid fever the saliva so vitiated as to soften the entire enamel surface. Three out of every four cases tested give acid reactions. The brown varieties of decay are due to sulphuric, the white to nitric acid. These are generated in the mouth. In gout and rhumatism, lactic acid is always present in the saliva, generated elsewhere in the system.

Dr. Chisholm—I do not believe that sulphuric and nitric acids are ever present in the saliva before it passes from the glands. When they are found in saliva, they arise from its oxidation. I have seen cases of chemical abrasion, but believe the acids come from the stomach, and not from the salivary glands. He who has healthy salivary glands has little to fear from decay, if he can keep the mouth free from foreign substances.

This subject occupied the society until the hour of adjournment.

AFTERNOON SESSION.

The society was called to order again at half-past 2 o'clock, and minutes of morning session were read and approved.

Dr. Morgan reported that a communication had been received from Dr. T. C. Edwards, of Brownsville, asking to be made a member of the Association.

Dr. Claywell spoke highly of Dr. Edwards, saying he was a gentleman of good moral character, and a graduate of the Baltimore Dental College; but the chair decided that he could not consistently with the rules of the society be made a member without being present.

The Committee on Dental Literature being called for, Dr. Morgan, Chairman, read a paper on that subject; in which he noticed all of the recent publications on dental surgery, and collateral subjects, including Wedl's "Dental Pathology," Bloxam's "Laboratory Teaching," Meridith's "How to Take Care of the Teeth," and Arthur's "Treatment and Prevention of Decay," He next proceeded to notice the periodical literature of the profession, urging upon members the importance of taking and supporting the journals; and showing how impossible it is to keep pace with the progress of dentistry without the use of these means. He proceeded to show up the defects in the dental periodicals of the day, but suggested that these are due as much to want of proper support from the prefession as to defective management.

The report of the Committee on Mechanical Dentistry was now called for, and Dr. Chisholm, Chairman, said he had no report, and thought the Society would not be interested in his method of mounting artificial teeth. Proposes to use celluloid in the place of rubber until the present monopoly is done away with.

Dr. Noel reported a case of failure with this base, owing to the shrinking of the material. Believes it will all shrink, and regards it as unfit for a base for artificial teeth.

Dr. Chisholm called attention to the practice of making deep suction cavities in plates as one that cannot be too much condemned. Puts his patients off with as little chamber as possible. Uses a piece of visiting card to make chambers in vulcanite plates.

Dr. Buckner—I concur with Dr. Chisholm in his views with regard to air chambers, but I think he ought to throw away his pasteboards. I use no air chamber at all, and have no difficulty.

Dr. Ross spoke of a case coming under his observationr in which not only the mucous membrane of the palate, but the palate bones, had conformed themselves to the shapes of the chamber.

This subject was discussed until the hour of adjournment.

Dr. Morgan was appointed to give a clinic to-morrow morning at his office, at half-past 8 oclock.

The Society now adjourned to meet at 10 A. M. to morrow.

SECOND DAY, MORNING SESSION.

At half-past 8 o'clock Dr. Morgan held a clinic at his office, and at 10 the Society met again at Dr. Ross' office. The attendance was now augmented by the addition of Drs. J. Taft, of Cincinnati, and W. G. Redman, of Louisville.

The Executive Committee offered the names of these gentlemen for membership, and upon balloting they were unanimously elected members of the Association.

The report of the Committee on Dental Chemistry was now called for, and Dr. Noel, Chairman of the committee, read a paper, in which the action of alkalies upon the teeth, and upon oxychloride of zinc fillings was discussed. (See another page of this Number Ed.]

Discussion being called for, Dr. Chisholm said that the ideas advanced in the paper fully accorded with his own views of the action of alkalies on the teeth, and of the durability of oxychloride of zinc fillings. Dr. Freeman has observed that oxychloride of zinc will frequently wash out of the superior teeth while it remains intact in the inferior teeth of the same mouth.

Prof. Taft commended the manner in which the writer of the paper had yielded his preconceived ideas to the force of demonstration. He was surprised at the views advanced by Prof. Somers in regard to the action of alkalies on the teeth and was inclined to think he had not investigated the matter by actual experiment. Those who are familiar with chemical reactions are liable to fall into this error, from the fact that the gelatinous portion of the tooth is dissolved out in most instances of decay. Acids also dissolve out the gelatinous portion in many cases. The experiments given show that much stronger alkalies were used than can ever exist in the mouth. It takes a very strong stretch of the imagination to suppose that alkalies can be the great agents in the production of decay. This subject is not as well understood as it ought to be. Investigation in this direction is calculated to do much good in the prevention of decay. He is not aware that anyone is now relying on oxychloride of zinc for permanent fillings. Certainly no one who claims to be at all acquainted with his profession. He referred to the variety of preparations in the market; the various modes of manipulating them; and the various degrees of solvent power existing in the fluids of the mouth. It is hard to determine the value of a material tested under so many modifying circumstances. Has seen it last for five years, and has seen it wash out in two months.

Dr. Chisholm wishes to correct a misunderstanding that occurred yesterday, in connection with some remarks he made concerning the function of enamel. He was understood to say that he had seen two surfaces of dentine in actual contact for twenty years without decay; an idea he did not wish to convey. Dr. Morgan calls attention to those cases where the enamel is left overhanging large cavities, showing that it resists disintegrating agents longer than dentine.

Dr. Herman thinks the gentlemen agree in the main, but cannot understand each other. The enamel is harder, and resists decay better; but both enamel and dentine are liable to decay.

Dr. Morgan—All that I have assumed, is that the enamel is a protection to the dentine.

Dr. Taft—I see little ground for difference of opinion on this subject. The increased density of enamel protects the dentine in two ways: first, from attrition; second, from the action of chemical agents. It does resist chemical action longer than dentine. It has also vitality. These two elements of resistence make it less vulnerable than dentine. The enamel is not only endowed with vitality but it receives a degree of nourishment. Where one point of defect is found in its microscopic structure, two or three are found in that of dentine. This enables decomposing agents to act more readily upon the latter. In the granular layer the vitality is very low, and it decays more rapidly than any other portion.

Dr. Morgan, has always regarded the enamel as vital, and thinks it receives its nourishment through the dentinal tubuli. Where this connection is cut off it dies. Calls attention to the importance of rubbing the gums with a napkin, so as to induce a healthy action of the mucous follicles.

Dr. Taft finds that the acidity of the mucous is increased in proportion to its viscidity. *Lactic* acid is the great destructive agent in the decay of the teeth. When the mineral acids are present they arise from decomposing nitrogenous substances.

The Executive Committee presented the name of Dr. B. B. Bushfield, for membership. He was unanimously elected a member of the Association. The Society now adjourned to meet at half-past 2 P. M.

SECOND DAY, AFTERNOON SESSION.

Persuant to adjournment the Society met at half-past 2 o'clock—President in the chair.

The Committee on Dental Ethics was called for. No report.

Dr. Chisholm—I am unwilling to pass this subject without having it discussed. To its neglect, in some way, is due the fact that so few are in attendance upon this meeting.

Dr. Morgan thought the subject not one prolific of discussion. Our code is a plain document, and one easily understood.

Dr. Taft—The code is liable to different interpretations. Some of the profession have made exhibition in public places of their workmanship, in the form of artificial dentures, filled teeth, etc. This has given rise to trouble in some quarters, and the question has been sprung as to whether this is a violation of the code. Would like to have an expression from the Association in regard to this matter.

Dr. Noel introduced the following resolution.

*Resolved:* That this society regards it as a violation of the code of ethics of the American Dental Asssociation, and not in accordance with true professional conduct for members of that body, or of organizations in which that code is adopted; to exhibit in fairs, expositions, or other public places, specimens of their workmanship, such as artificial dentures, filled teeth, etc.

Drs. Morgan, Taft, and Redman spoke in strong condemnation of this custom.

Dr. Freeman—I favor the resolution, and under the circumstances it requires a deal of moral courage to do so, being myself one of the exhibitors.

Dr. Taft commended Dr. Freeman highly for the position he was about to take. Thought he ought not to feel badly about it. If he finds himself in the wrong, he ought to get right as quickly as possible.

Dr. Freeman—That is the way I feel about it, therefore I am in favor of the resolution.

The question was now put, and the resolution unanimously adopted.

The subject of ethics occupied the Society the remainder of the afternoon.

Adjourned to meet at half-past seven o'clock.

SECOND DAY, EVENING SESSION.

At half-past 7 o'clock the Society was called to order, and the report of the Committee on Dental Edncation was called for, (this committee was appointed in 1871, to prepare a treatise for popular reading on, "The teeth, their structure, develment, and physiological importance." It was the object of the society to answer many of the questions arising in the popular mind, and to give such simple instructions as to the care of the teeth, as the public at large stand in need of.) The committe failing to report, a voluntary treatise on that subject was offered, and read.

Dr. Freeman thought the paper a good one and a credit to the Society, but thought it was not applicable to the class of people the society designs to reach. Some of the members objected to its length, some to minor points in its teaching, and some thought it employed too many technicalities; but all agreed that it could be so revised and corrected as to meet the wants of the Society.

Dr. Redman thought it was the best paper he had ever heard on that subject, but thought it a little too lengthy, and suggested that it be shortened, by leaving out the chapter on artificial teeth.

Dr. Taft had seen good results come of distributing such works. He refered to the good that had been accomplished, by the circulation of "The Dental Luminary," published in Cincinnati. He was pleased with the paper, and thought the Society ought to proceed at once to publish it. He recommended that the proof sheets be sent to the various members for correction and suggestion. Then he thought it was best to have the work stereotyped.

Dr. Morgan moved that the paper be adopted, and submitted to a committee, who shall correct and publish it, drawing on the Society for funds. The motion was carried.

A committee was elected, consisting of the following members: W. H. Morgan, R. R. Freeman, and L. G. Noel.

On balloting for place of next meeting Nashville was chosen.

The President was authorized to appoint delegates to the various dental associations. This being an adjourned meeting, the present incumbents were continued as officers for the ensuing year.

Dr. Freeman now read a paper on "Filling Teeth."

A very lengthy and spirited discussion of the subject ensued, during the course of which reference was made to Dr. Mack's gold screws for retaining fillings; when Dr. Morgan spoke in high terms of the method. He thought the credit only of perfecting the practice due to Dr. Mack, as it was described by W. H. Dwinell, as far back as 1845, in the *American Journal of Dental Science.*

The hour being late, the minutes of the session were read and approved, and the Association adjourned, to meet in this city, on the fourth Wednesday in Oct., 1873.

L. G. Noel, Recording Secretary.

